# H-Shaped Vertebrae

**DOI:** 10.5334/jbsr.3234

**Published:** 2023-09-14

**Authors:** Annelien Massart, Gert-Jan Allemeersch

**Affiliations:** 1 UZ Brussel, BE; 2 Radiology Department, UZ Brussel, BE

**Keywords:** H-shaped vertebra, Lincoln log vertebrae, bony lesions sickle cell disease

## Abstract

**Teaching Point:** H-vertebrae are pathognomonic for sickle cell disease.

## Case History

A 37-year-old woman, known to have homozygous sickle cell disease, was referred for magnetic resonance imaging (MRI) of the lumbar spine for worsening lower back pain. She had been under treatment for the disease since 2007, with hydrea and a folic acid supplement. Over the years she had suffered multiple vaso-occlusive crises of an acute chest syndrome.

**Figure 1:**
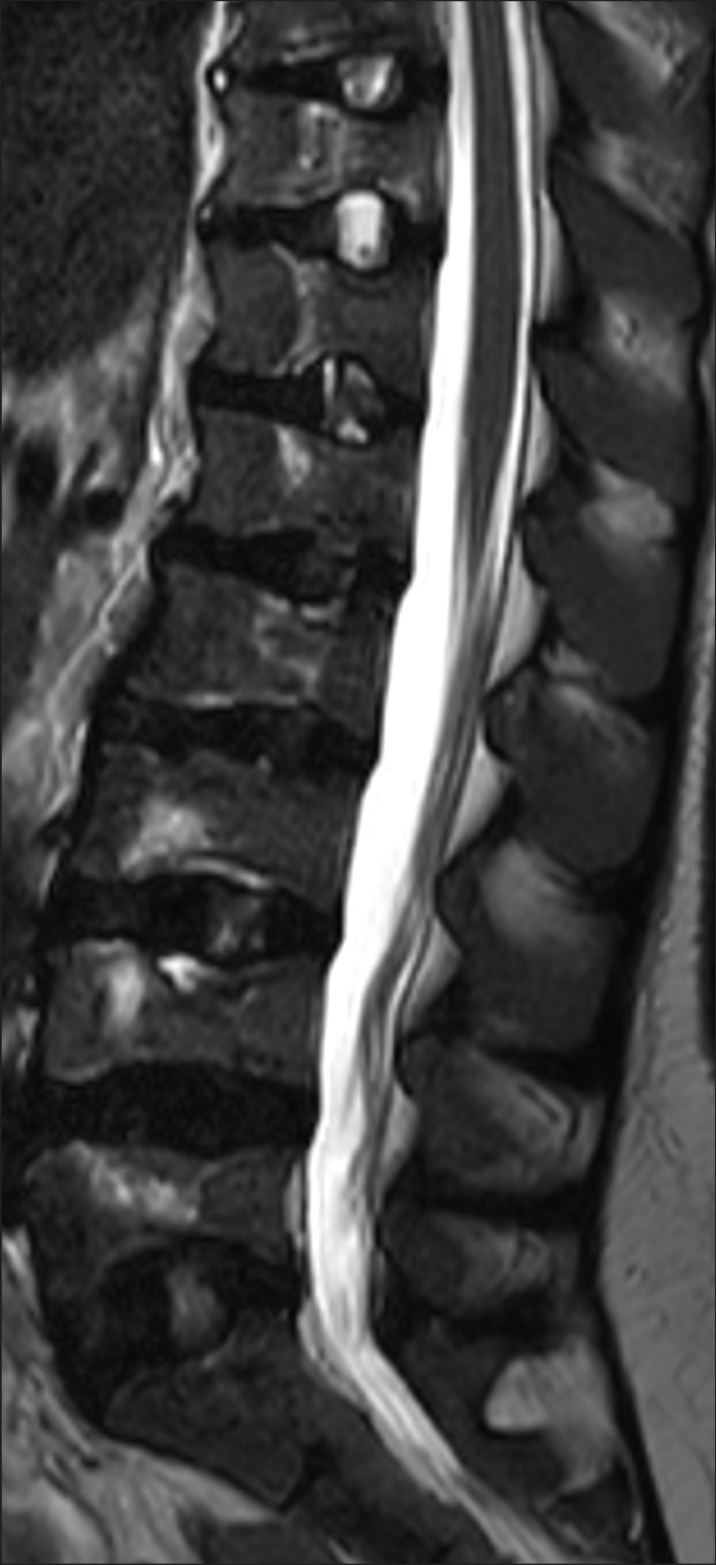


The MRI revealed a central depression of consecutive vertebral endplates in the lumbar spine on the sagittal images, which have a typical H-shape, also called the Lincoln log vertebrae (Figure 1). This square-shaped central depression of the end plate of the vertebrae is caused by the microvascular endplate infarction with a secondary overgrowth of the surrounding portions. A second theory states that the periphery of the endplates is spared because of collateral circulation.

**Figure 2:**
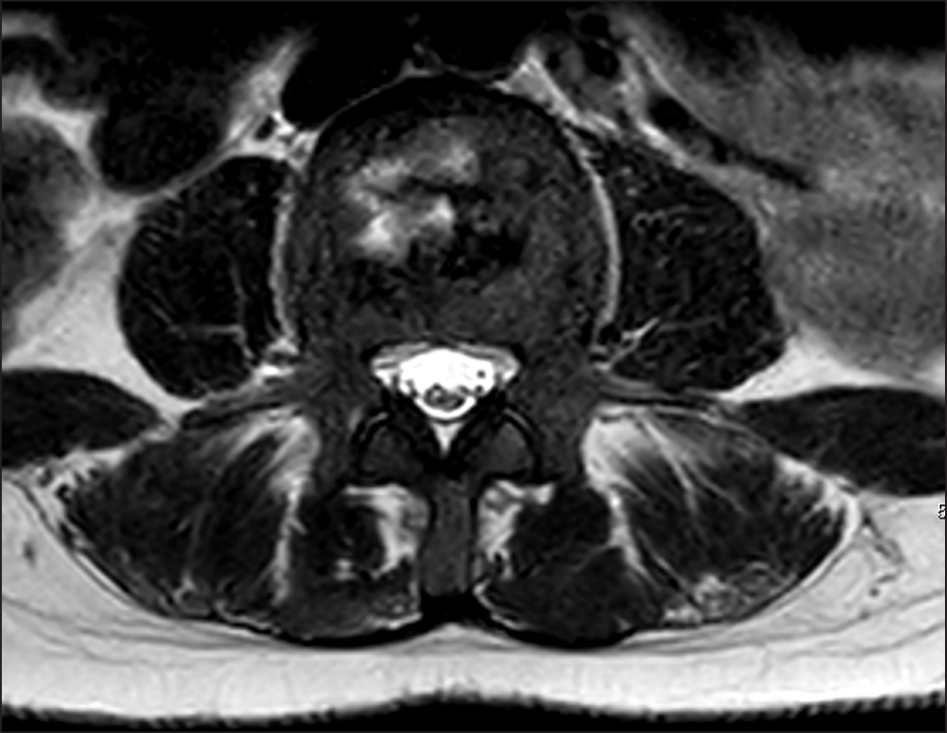


MRI also showed multiple T2 hyperintense central lesions in several vertebral bodies, which represent bone marrow edema due to bone infarctions (Figures 1 and 2).

## Comments

Sickle cell disease is an inherited blood disorder characterized by abnormal hemoglobin, leading to the formation of sickle-shaped red blood cells. This condition is prevalent in individuals of African, Middle Eastern, and Mediterranean descent, with an estimated 100,000 people affected in the United States alone [1].

Patients with sickle cell anemia can present with a wide range of symptoms and complications, including pain crises, acute chest syndrome, stroke, and infections, among others.

Sickle cell anemia is caused by a point mutation in the genetic coding for the b chain of hemoglobin, leading to the production of abnormal hemoglobin molecules which tend to clump together and form large, rigid polymer strands within the red blood cells, creating elongated (sickle-shaped) red blood cells with less deformability, which can in turn obstruct blood flow. In addition, the red blood cells in sickle cell disease show a higher binding affinity for the vascular endothelium, worsening the vascular occlusion leading to tissue ischemia and infarction. The abnormally shaped red blood cells in turn are removed from the blood at higher rates causing hemolytic anemia [1].

These vaso-occlusive crises are responsible for bone marrow infarction and subsequent bone pain at the level of the skeletal system. Infarction typically takes place in the medullary cavity and the epiphysis but can occur in every marrow containing bone. In the spine, it gives rise to H-shaped vertebrae also known as the Lincoln log vertebrae, which is pathognomonic for sickle cell disease and can be seen in 10% of patients [1].
